# Quercetagitrin Inhibits Tau Accumulation and Reverses Neuroinflammation and Cognitive Deficits in P301S-Tau Transgenic Mice

**DOI:** 10.3390/molecules28093964

**Published:** 2023-05-08

**Authors:** Suyue Zhong, Jinwang Ye, Yunsong Deng, Mohan Zhang, Miaozhan Zou, Xuanbao Yao, Shifeng Xiao

**Affiliations:** 1 Shenzhen Key Laboratory of Marine Biotechnology and Ecology, College of Life Sciences and Oceanography, Shenzhen University, Shenzhen 518060, China; doravzsy@foxmail.com (S.Z.);; 2 School of Chemistry and Chemical and Engineering, Guangxi Minzu University, Nanning 530008, China; 3 Shenzhen-Hong Kong Institute of Brain Science-Shenzhen Fundamental Research Institutions, Shenzhen 518055, China

**Keywords:** tau protein, quercetagitrin, inflammation, gliosis, memory

## Abstract

Intracellular tau accumulation is a hallmark pathology of Alzheimer’s disease (AD) and other tauopathies. Tau protein, in the hyperphosphorylated form, is the component of paired helical filaments (PHFs) and neurofibrillary tangles (NFTs) in AD. Blocking tau aggregation and/or phosphorylation is currently a promising strategy for AD treatment. Here, we elucidate that quercetagitrin, a natural compound derived from African marigold (*Tagetes erecta*), could inhibit tau aggregation and reduce tau phosphorylation at multiple disease-related sites in vitro. Moreover, the in vivo effect of quercetagitrin was assessed in P301S-tau transgenic via oral administration. The compound treatment restored the cognitive deficits and neuron loss in the mice. The formation of NFTs and tau phosphorylations in the hippocampus and cortex of the mice was also prevented by the compound. Moreover, quercetagitrin feeding displayed neuroinflammation protection through the inhibition of NF-κB activation in the mice. Together, our data reveal that quercetagitrin possesses the potential to further develop as a therapeutic medicine for AD and other tauopathies.

## 1. Introduction

Tau, a microtubule-associated protein, is enriched in the brain and mainly localized in neuronal axons. It plays an important role in microtubule assembly and stabilization. There are six major isoforms of tau in the human brain (including N-terminal 0–2 N and C-terminal 3/4R), which are formed by alternative splicing of the MAPT gene in chromosome 17 (17q21-q22) [1] In physiological conditions, the native tau is hypophosphorylated and shows little tendency for accumulation. However, hyperphosphorylated tau is the primary component of paired helical filaments (PHFs) and neurofibrillary tangles (NFTs), characterizing a wide range of neurodegenerative diseases known as tauopathies [2].

Tauopathies are consistent with a variety of neurodegenerative diseases including frontotemporal dementia, Parkinson’s disease, and Alzheimer’s disease (AD), the most common form of dementia. In neurodegenerative diseases with tauopathy, the oligomeric tau aggregates are believed to act as the most neurotoxic species and dominate the major aspects of tau-induced neuropathology. In wild-type mice, subcortical stereotaxic injection of recombinant tau oligomers impairs the memory and induces synaptic and mitochondrial dysfunctions [3]. In mice overexpressing mutated α-synuclein (A53T mice), treatment with a tau oligomer-specific monoclonal antibody efficiently protects the mice from cognitive and motor deficits with decreased toxic tau oligomers levels. Moreover, tau oligomer depletion also protects against dopamine and synaptic protein loss [4]. However, experimental evidences from post-mortem human brain and various animal and cell models have suggested that oligomeric tau undergoes cell-to-cell transmission, which raises concern about the long-term beneficial effect of anti-tau antibody immunotherapy [5]. In order to reduce tau pathology in AD, a variety of small molecules have been described, including modulators of post-translational modifications and aggregation inhibitors, most of which are in the preclinical stage [6].

Flavonoids are types of polyphenolic phytochemicals mostly found in fruits, vegetables, and nuts, presenting multiple pleiotropic properties (they act on different cellular targets and biochemical mechanisms). Many studies describe the multiple mechanisms of the neuroprotective activity of flavonoids [7]. Quercetagitrin, also known as quercetagetin-7-O-glucoside, is a flavonoid derivative extracted from marigold (*Tagetes erecta*). The anti-inflammatory effects of the compound have been reported in multiple inflammatory diseases. In rat neutrophils, quercetagitrin yielded more than 60% of inhibition on lysosomal enzyme secretion and arachidonic acid release [8]. In HaCaT human keratinocytes, quercetagetin effectively blocked IFN-γ and TNF-α-induced chemokines CCL17 and CCL22 expression [9]. However, the role of quercetagitrin in neurodegenerative diseases, especially tauopathy, remains unclear. Here, for the first time, we investigate the therapeutic effects of quercetagitrin on tau pathology. Our results show that quercetagetin could inhibit tau aggregation and tau phosphorylation both in vivo and in vitro, with attenuated NF-κB activation, neuroinflammation, neuronal loss, synaptic impairments, and cognitive deficits in the P301S-tau transgenic mouse model. The results here provide in vivo evidence for further development of quercetagetin as potential drug candidate for AD and other tauopathies.

## 2. Results

### 2.1. Quercetagitrin Blocks Tau Aggregation

**Figure 1:**
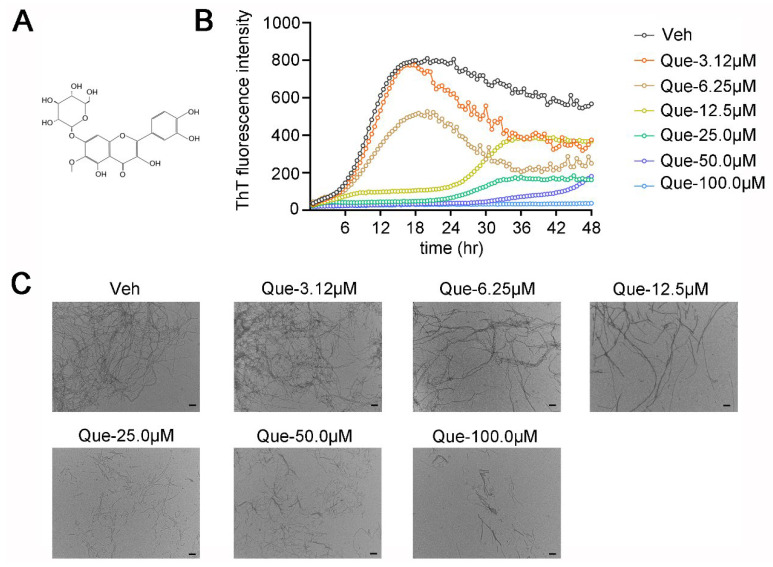
Quercetagitrin inhibits tau aggregation in vitro. (**A**) Structure of quercetagitrin. (**B**) Heparin-induced fibrillization of tau-K18 protein monitored by ThT fluorescence in the presence of different concentrations of quercetagitrin (0, 3.12, 6.25,12.5, 25.0, 50.0, and 100 μM). (**C**) Representative TEM images of tau-K18 aggregation products with different concentration of quercetagitrin or control vehicle (scale bar = 0.2 μm).

To explore the effect of quercetagitrin on the tau aggregation process, an in vitro heparin-induced tau-K18 aggregation was performed via the ThT fluorescence assay. In the control group, tau-K18 started to aggregate at 6 h, and the ThT fluorescence reached its peak at 18 h, while in the low concentration quercetagitrin-treated groups (3.12 and 6.25 μM), quercetagitrin decreased the peak fluorescence but did not affect the aggregation time. In the high concentration quercetagitrin-treated groups (12.5, 25, 50, and 100 μM), besides the peak fluorescence, the aggregation start time was pushed back to more than 24 h (Figure 1B). These results suggested that quercetagitrin reduced the amount of β-sheet structure in the protein aggregates.

To make the fluorescence results more intuitive and convincing, transmission electron microscopy (TEM) imaging was utilized to observe the morphology of tau-K18 fibrils. The mature tau-K18 fibrils in the control group could be described as long and compact fibrils in contrast to those in the quercetagitrin-treated groups. Quercetagitrin inhibited tau aggregation in a dose-dependent manner, as shown by the reduced tau fibrillization (Figure 1C). These results suggest that quercetagitrin is able to reduce tau aggregation through the blocking of tau fibrillization.

### 2.2. Quercetagitrin Decreases Tau Phosphorylation Levels in HEK293 Cells and N2a Cells

**Figure 2:**
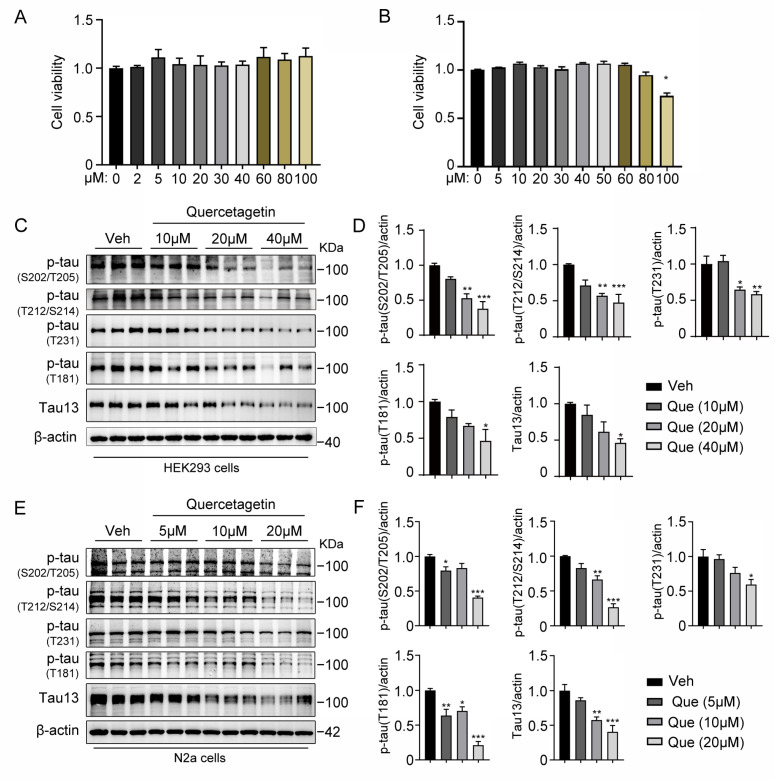
Quercetagitrin decreases tau phosphorylation in HEK293 cells and N2a cells. (**A**,**B**) The viability of HEK293 cells (**A**) and N2a cells (**B**) was determined by CCK-8 assay in the presence of gradient concentration of quercetagitrin. N = 4 for each group, unpaired Student’s *t* test. (**C**,**D**) HEK293 cells were transfected with human 1N4R P301S-tau plasmids for 24 h and then incubated with quercetagitrin for another 24 h. (**C**) Representative western blots of AT8 (Ser202/Thr205), AT100 (Thr212/Ser214), AT180 (Thr231), AT270 (Thr181), and total tau (tau13). (**D**) Quantification analysis of phosphorylated tau and total tau levels normalized to GAPDH. N = 3 for each group, one-way ANOVA, Dunnett’s post hoc analysis. (**E**,**F**) N2a cells were transfected with human 1N4R P301S-tau plasmids for 24 h and then incubated with quercetagitrin for another 24 h. (**E**) Representative western blots of AT8 (Ser202/Thr205), AT100 (Thr212/Ser214), AT180 (Thr231), AT270 (Thr181), and total tau (tau13) are shown. (**F**) Quantification analysis of phosphorylated tau and total tau levels normalized to β-actin. N = 3 for each group, one-way ANOVA, Dunnett’s post hoc analysis. Data were expressed as mean ± SEM, * p < 0.05, ** p < 0.01, *** p < 0.001.

Apart from aggregation, hyperphosphorylation is another critical tau pathology. To investigate the effect of quercetagitrin on tau phosphorylation, we applied it in tau-overexpression HEK293 cells and N2a cells. Firstly, we evaluated the cytotoxicity of quercetagitrin and found no significant impairments of cell viability (up to 100 μM) in both HEK293 cells (Figure 2A) and N2a cells (Figure 2B). Next, GFP-fused 1N4R P301S-mutant human tau was transfected and overexpressed in HEK293 cells and N2a cells, and then the cells were treated with quercetagitrin. Western blotting analysis showed that the compound decreased tau phosphorylation levels in a concentration-dependent manner, as shown by reduced AT8 (Ser202/Thr205), AT100 (Thr212/Ser214), AT180 (Thr231), and AT270 (Thr181) levels in both cells (Figure 2C–F). Moreover, decreased total tau levels were also observed in the quercetagitrin-treated cells (Figure 2C–F). The consistent efficiency of quercetagitrin in both neuronal cells (N2a cells) and non-neuronal cells (HEK293 cells) implies that it is a potentially biologically active drug that antagonizes tau phosphorylation and accumulation.

### 2.3. Quercetagitrin Reduces Tau Phosphorylation and Accumulation in P301S-Tau Transgenic Mice

**Figure 3:**
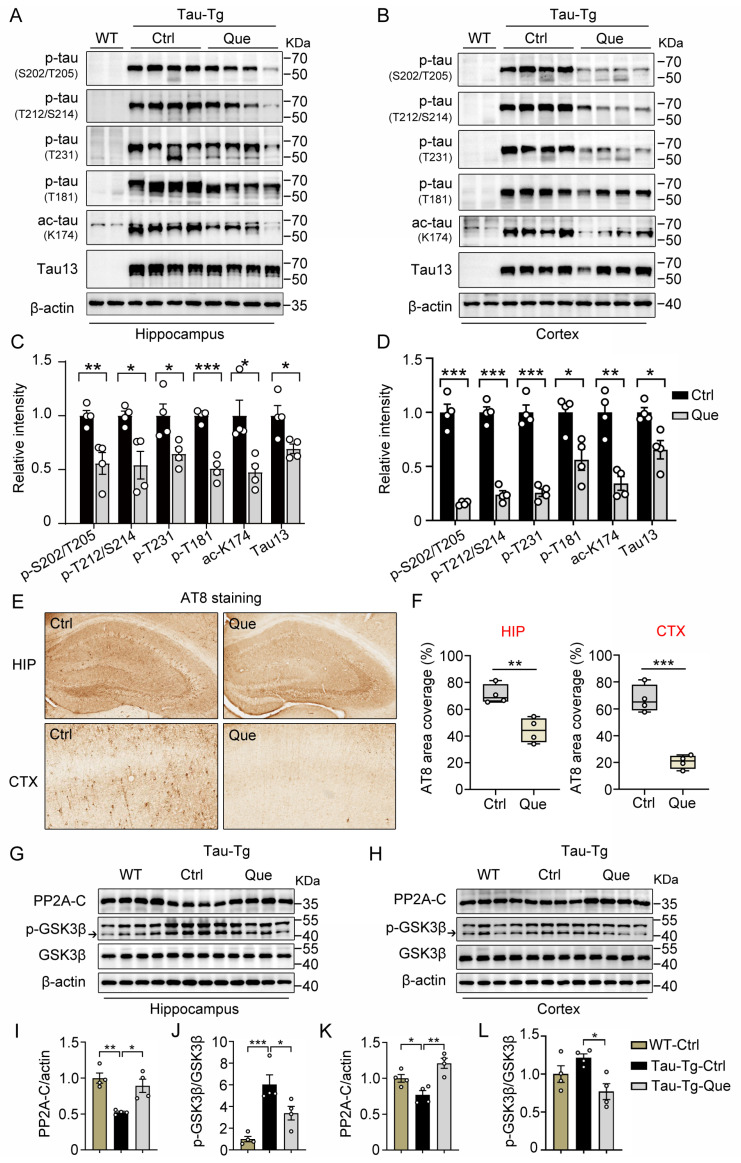
Quercetagitrin reduces tau phosphorylation in P301S-tau transgenic mice. After feeding with quercetagitrin for three months, tau pathology in nine-month-old male P301S-tau transgenic mice was analyzed. (**A**,**B**) Representative western blots of AT8 (Ser202/Thr205), AT100 (Thr212/Ser214), AT180 (Thr231), AT270 (Thr181), ace-tau(K174), and total tau (tau13) in the hippocampus (**A**) and cortex (**B**). (**C**,**D**) Quantification analysis of phosphor-tau, ace-tau and total tau levels in hippocampus normalized to GAPDH (**C**) and cortex normalized to β-actin (**D**). N = 4 for each group, unpaired Student’s *t* test. (**E**) Representative immunohistochemical images of anti-AT8 antibody staining in the hippocampus (upper) and cortex (lower) of P301S-tau transgenic mice (scale bar: 100 μm). (**F**) Quantification analysis of normalized AT8 positive area in the hippocampus and cortex. N = 4 for each group, unpaired Student’s *t* test. (**G**–**L**) Western blot analysis of PP2A-C, p-GSK3β(Y216), total GSK3β, and β-actin in the hippocampus (**G**) and cortex (**H**) of P301S-tau transgenic mice treated with quercetagitrin or vehicle. (**I**–**L**) Quantification analysis of PP2A-C normalized to β-actin and p-GSK3β(Y216) normalized to total GSK3β. N = 4 for each group, one-way ANOVA, Dunnett’s post hoc analysis. Data were expressed as mean ± SEM, * p < 0.05, ** p < 0.01, *** p < 0.001.

We next tested whether quercetagitrin could improve tau pathologies in vivo. The P301S-tau transgenic mouse, an excellent animal model for tauopathies, develops prominent tau pathology and cognitive deficits at six months old. Here, six-month-old male P301S-tau transgenic mice were treated with quercetagitrin (10 μg/mL) for three consecutive months through water feeding. Western blotting analysis showed significantly reduced tau phosphorylation levels both in the hippocampus and the cortex of the mice, as manifested by decreased levels of AT8 (Ser202/Thr205), AT100 (Thr212/Ser214), AT180 (Thr231), and AT270 (Thr181) (Figure 3A–D). Interestingly, a remarkably decreased acetylation level at Lys174 was found both in the hippocampus and the cortex as well (Figure 3A–D). The total human tau level (recognized by Tau13 antibody) was slightly reduced in the cortex and remained unchanged in the hippocampus (Figure 3A–D). Furthermore, immunohistochemistry staining of anti-AT8 antibody also revealed a reduced tau accumulation in the two brain areas of quercetagitrin-treated mice (Figure 3E,F).

The protein phosphatase 2A (PP2A) and the glycogen synthase kinase 3β (GSK3β) play critical roles in regulating tau phosphorylation [10]. Therefore, we measured the expression levels or the activity-dependent changes of PP2A and GSK3β. Reduced PP2A-C levels and increased phospho-GSK3β levels at Y216 are found in P301S-tau transgenic mice compared to WT mice. Expectably, quercetagitrin treatment significantly increased PP2A-C levels in both brain regions and prevented augmented GSK3β activity in the hippocampus (Figure 3G–L). These results demonstrate that quercetagitrin could effectively reduce tau phosphorylation through the regulation of PP2A and GSK3β.

**Figure 4:**
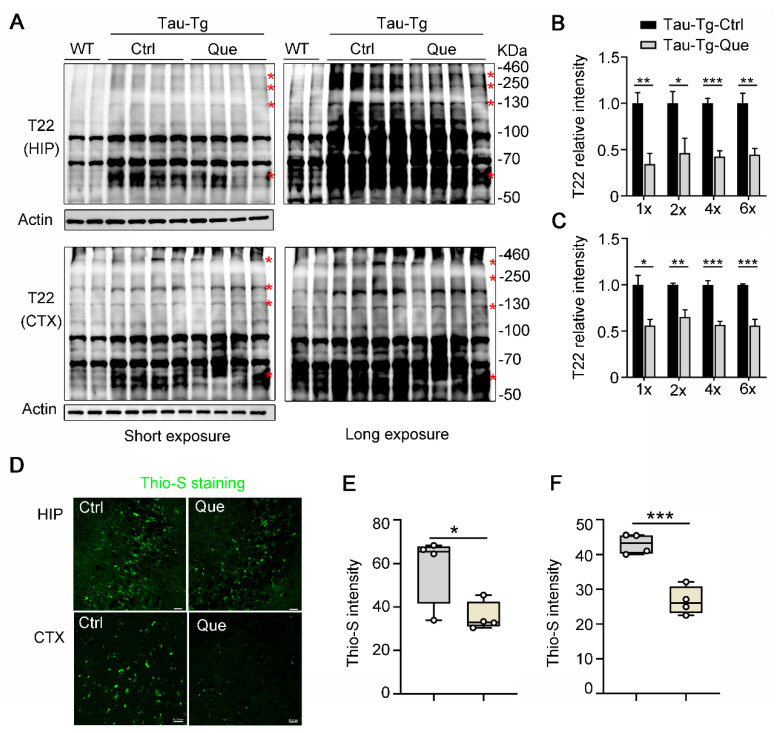
Quercetagitrin diminishes tau oligomerization and tau fibrillization in P301S-tau transgenic mice. (**A**) The oligomeric tau (~130 KDa, 250~460 KDa) in the hippocampus and cortex of P301S-tau transgenic mice treated with quercetagitrin or vehicle was measured by means of western blotting using anti-T22 antibody (which specifically reacts with oligomeric tau). (**B**,**C**) Quantification analysis of oligomeric tau (1×, 2×, 4× and 6×) in the hippocampus (**B**) and cortex (**C**). N = 4 for each group, unpaired Student’s *t* test. (**D**) Representative Thio-S staining images in the brain of control and quercetagitrin-treated P301S-tau transgenic mice (microscope: ZEISS LMS 880, object lens: 40×, scale bar: 20 μm). (**E**,**F**) Thio-S intensity analysis in the hippocampus (**E**) and cortex (**F**). N = 4 for each group, unpaired Student’s *t* test. Data were expressed as mean ± SEM, * p < 0.05, ** p < 0.01, *** p < 0.001.

Gradually persistent tau hyperphosphorylation promotes tau oligomerization and fibrillization, which build up the formation of PHFs and NTFs in AD [10]. Tau oligomers and fibrils within the mouse brain were measured by western blot analysis using an anti-T22 antibody (which specifically reacts with oligomeric tau) and thioflavin-S staining, respectively. Quercetagitrin treatment effectively reduced tau oligomerization (Figure 4A–C) and fibrillization (Figure 4D–F) both in the hippocampus and the cortex. Taken together, these results demonstrate that quercetagitrin is a promising compound in attenuating tau pathologies in P301S-tau transgenic mice.

### 2.4. Quercetagitrin Treatment Reverses Neuroinflammation and Neuronal Loss in P301S-Tau Transgenic Mice

**Figure 5:**
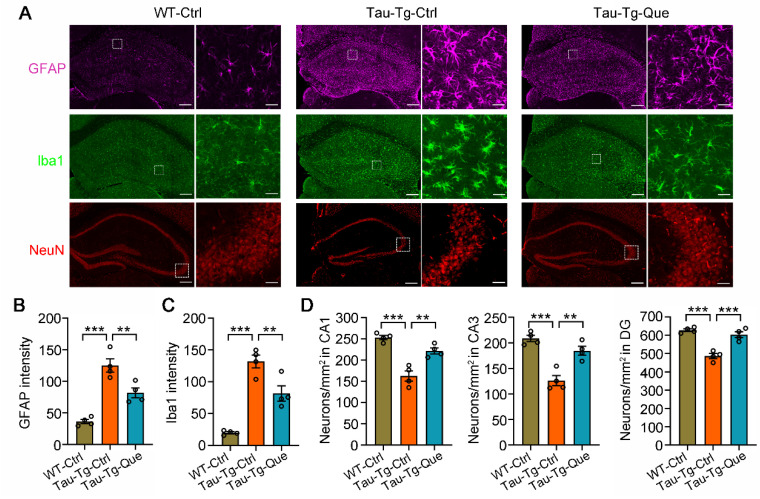
Quercetagitrin treatment reverses gliosis and neuronal loss in P301S-tau transgenic mice. (**A**) Representative immunofluorescence images of astrocyte marker (GFAP), microglia marker (Iba1), and neuron marker (NeuN) in the hippocampus of WT mice and control or quercetagitrin-treated P301S-tau transgenic mice (microscope: ZEISS LMS 880, object lens: 10×, scale bar: 200 μm for the whole hippocampal images and 20 μm for the enlarged images). (**B**,**C**) Quantification analysis of the GFAP fluorescence intensities (**B**) and Iba1 fluorescence intensities (**C**) in the hippocampus of P301S-tau transgenic mice. N = 4 for each group, one-way ANOVA, Dunnett’s post hoc analysis. (**D**) Quantification analysis of neuron numbers in the hippocampal CA1, CA3, and DG subset, respectively. N = 4 for each group, one-way ANOVA, Dunnett’s post hoc analysis. Data were expressed as mean ± SEM, ** p < 0.01, *** p < 0.001.

Having demonstrated the efficacy of quercetagitrin in reducing tau aggregation, we tested whether quercetagitrin could reverse tau pathology-related neuroinflammation, glia cell activation, and neuronal damage in vivo. Co-immunofluorescence staining experiments using an astrocyte marker (GFAP), a microglia marker (Iba1), and a neuron marker (NeuN) were carried out in wild-type C57B6 mice and P301S-tau transgenic mice treated with vehicle or quercetagitrin. The results showed that quercetagitrin treatment prominently limited astrogliosis and microglia activation in the hippocampus of P301S-tau transgenic mice, as indicated by a reduced GFAP and Iba1 expression intensity (Figure 5A–C). The activation of nuclear factor-kappa B (NF-kB) signaling is closely associated with tau pathology via neuroinflammation [11]. Western blot analysis displayed markedly decreased NF-kB phosphorylation and mitigated inflammatory responses, as shown by reduced Il-1α and Il-6 protein levels, both in the hippocampus and cortex (Supplementary Figure S1). Neuronal loss in the six-month-old P301S-tau transgenic mice was also restored upon quercetagitrin administration, as shown by increased neuron numbers in the hippocampal CA1, CA3, and DG subsets (Figure 5A,D).

### 2.5. Quercetagitrin Treatment Attenuates Synaptic Impairments and Cognitive Deficits in P301S-Tau Transgenic Mice

**Figure 6:**
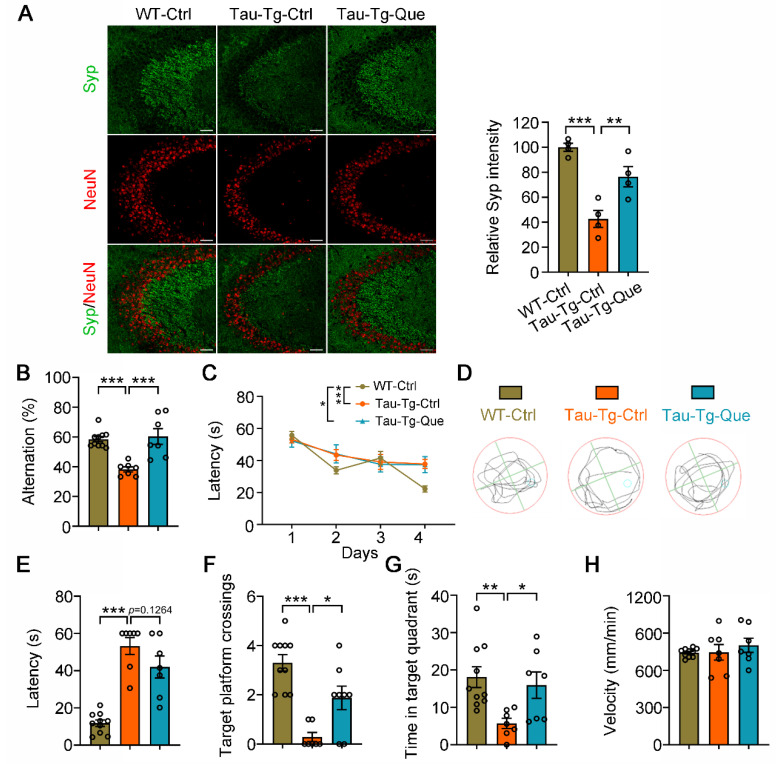
Quercetagitrin treatment attenuates synaptic impairments and cognitive deficits in P301S-tau transgenic mice. (**A**) Representative immunofluorescence images showing expression levels of synaptophysin and NeuN in the hippocampal CA3 subset of WT and control or quercetagitrin-treated P301S-tau transgenic mice (microscope: ZEISS LMS 880, object lens: 20×, scale bar: 50 μm). Quantification analysis shows the relative synaptophysin fluorescence intensity in hippocampal CA3 subset. N = 4 for each group, one-way ANOVA, Dunnett’s post hoc analysis. (**B**) Quercetagitrin treatment restores spatial working memory in the Y-maze test. Quantification of the alternation of WT and control or quercetagitrin-treated P301S-tau transgenic mice in the Y-maze test. N = 7–10 for each group, one-way ANOVA, Dunnett’s post hoc analysis. (**C**–**H**) Quercetagitrin treatment attenuates cognitive deficits measured by Morris water maze (MWM). (**C**) The learning latency in the training phase. (**D**) The representative move trajectories of the mice. The quantification of latencies to reach the target platform region (**E**), target platform crossings (**F**), and time spent in the target quadrant (**G**) measured at day 6 after removing the hidden platform. (**H**) The swimming speed of the mice in the three groups. N = 7–10 for each group, one-way ANOVA, Dunnett’s post hoc analysis. Data were expressed as mean ± SEM, * p < 0.05, ** p < 0.01, *** p < 0.001.

Tau accumulation could lead to synaptic plasticity impairments and cognitive decline [10,12]. Thus, we performed a co-immunostaining analysis of the presynaptic protein synaptophysin with the neuronal marker NeuN in the hippocampal CA3 subset of vehicle- or quercetagitrin-treated P301S-tau transgenic mice and control C57B6 mice. The results showed that quercetagitrin treatment partially elevated synaptophysin expression in the tau transgenic mouse (Figure 6A). To determine the effects of quercetagitrin on cognitive function, a Y-maze test and a Morris water maze (MWM) test were performed on P301S-tau transgenic mice treated with vehicle or quercetagitrin and age-matched wild-type C57B6 mice. The vehicle-treated P301S-tau transgenic mice exhibited prominent learning and memory deficits compared to the control wild-type mice. In the Y-maze test, the impaired alternation score in P301S-tau transgenic mice was restored by quercetagitrin treatment (Figure 6B). In the MWM test, quercetagitrin treatment could not shorten the learning latency in the training phase (Figure 6C). Spatial memory was evaluated at day 6 after removing the platform (Figure 6D). As expected, quercetagitrin treatment efficiently ameliorated the memory deficits in P301S-tau transgenic mice, evidenced by decreased latency in reaching the target platform region (Figure 6E); increased target platform crossings (Figure 6F); and elevated exploring time in the target quadrant (Figure 6G). The swimming speeds among the three groups are comparable (Figure 6H). These data demonstrate that quercetagitrin treatment reduced gliosis, neuronal loss, synaptic impairments, and cognitive deficits in P301S-tau transgenic mice.

## 3. Discussion

Tau pathology is characterized by intracellular neurofibrillary tangles composed of hyperphosphorylated tau protein, which is identified as a leading cause of multiple neurodegenerative diseases, including the most common form of dementia, Alzheimer’s disease. The hyperphosphorylated tau protein promotes its detachment from microtubules, increases cytoplasmic tau levels, and boosts PHFs and NFTs formation [13]. Hyperphosphorylation of tau at Thr181, Ser202, Thr205, Thr212, Ser214, and Thr231 is found in the degenerating neurons of the AD brain during embryonic and early postnatal periods [1]. In the current study, we demonstrate that quercetagitrin could reduce tau phosphorylation at these sites in HEK293 cells and N2a cells and in P301S-tau transgenic mice. The underlying mechanism of quercetagitrin-mediated tau-aggregation inhibition could be attributed to the interaction between its aromatic structure and the hydrophobic β-sheet secondary structure of tau aggregates. Our previous studies have demonstrated that another structurally similar flavonoid, iso·bavachalcone, could directly interact with tau-K18 through binding sites I278 and V309 positioned in the R2 (VQIINK) and R3 (VQIVYK) of the protein and therefore exhibit an anti-aggregation effect [14,15]. Interestingly, we also noticed that quercetagitrin treatment could reduce tau acetylation at Lys174 in the P301S-tau transgenic mice. This is in accordance with previous studies showing that increased tau acetylation at Lys174 or Lys280 could promote tau accumulation and aggravate tau-mediated neurodegeneration and cognitive impairments [16,17].

Neuroinflammation and gliosis occur in early AD and other tau pathology-related neurodegenerative diseases and persist throughout the disease development. Those pathologies would encourage the formation of an inflammatory neuron–glia microenvironment and exacerbate tau-mediated synaptic impairment and neurodegeneration. Our results suggest that quercetagitrin treatment leads to attenuated hippocampal astro- and microgliosis, inhibited NF-κB signaling activation, and reduced the production of the inflammatory cytokines Il-1α and Il-6 in P301S-tau transgenic mice. NF-κB signaling activation is closely associated with neuroinflammation both in astrocytes and microglia, and it plays a critical role in the progression of tauopathy [11,18]. Quercetagitrin, isolated from the flowers of the African marigold (*Tagetes erecta*), has been reported to have an anti-inflammation effect in neutrophils [8,19]. Moreover, studies have indicated that quercetagetin, a similar flavonoid also derived from marigolds, could suppress NF-κB activation via the inhibition of c-Jun NH_2_-terminal kinases [20]. The inactivation of NF-κB signaling reduces astrogliosis, microgliosis, and inflammatory responses, and it protects against spatial memory deficits in WT and P301S-tau transgenic mice [11,18]. Thus, the reversed gliosis and inflammatory responses observed in P301S-tau transgenic mice might be attributable to the inhibitory effect of quercetagetin on NF-κB signaling.

Synaptic impairment is closely correlated with cognitive deficits in tau pathology [12,21]. Synaptophysin is a presynaptic vesicle membrane protein with major functions in regulating vesicle formation and release [22]. Reduced synaptophysin protein levels have been implicated in AD, Parkinson’s disease, and frontotemporal dementia. Impaired synaptic plasticity with decreased synaptophysin expression in the hippocampal CA3 subset has been observed in P301S-tau transgenic mice [23]. Our results revealed that quercetagitrin significantly attenuated neuronal loss and cognitive deficits, with restored synaptophysin level in the brains of P301S-tau transgenic mice.

In recent years, much of the literature has identified the tight correlation between tau pathology and the clinical progression of AD, and therapeutic strategies aimed at targeting toxic tau proteins are increasingly recognized as promising approaches [24,25,26]. Our findings suggest that quercetagitrin could lessen tau pathology by restoring memory function by blocking toxic tau formation and inhibiting NF-κB-related inflammatory responses. Other flavonoids such as quercetin and myricetin have also been found to have the potential to improve AD. A study showed that quercetin protects cognitive and emotional function in aged 3xTg-AD mice [27]. Myricetin and dihydromyricetin have been demonstrated to reverse AD pathologies by interacting with Aβ [28,29]. However, their pharmacological properties and bioavailability might place limitations on their application. Low bioavailability has always been one of the main limitations in developing flavonoids as oral medications. Research has illustrated that the glycoside form of flavonoid compounds has better absorption when taken orally [30]. With regard to its various neuroprotective effects, we believe that quercetagitrin is a potential drug candidate for AD and that further investigation in amyloidosis will be beneficial.

## 4. Materials and Methods

### 4.1. Expression and Purification of Tau-K18 Protein

Recombinant human tau-K18 (Q244-E372) proteins were extracted from *E. coli* BL-21 cells [14]. Briefly, BL-21 cells transfected with recombinant vector (tau-K18) were cultured for 12–16 h in LB medium containing 100 μg/mL ampicillin at 180 rpm, at 37 °C. Then, the cells were collected by centrifugation (6000× *g* rpm, 15 min), and were lysed by sonication (5 s sonication, 2 s interval, total 15 min) in bacterial lysate with 1 mM PMSF. The tau-K18 protein was purified by anion exchange chromatography (HiPrep CM FF 16/10, Uppsala, Sweden) and agarose chromatography (Hiload 16/600 Superdex 75 pg, Uppsala, Sweden) sequentially. A fast protein liquid chromatography system (AKTA, GE Healthcare, Boston, MA, USA) was used in this study. Purified fractions were lyophilized and identified by Coomassie brilliant blue staining. For anion exchange chromatography, start buffer: 25 mM Tris-HCl, 20 mM NaCl, pH 8; elution buffer: 25 mM Tris-HCl, 1 M NaCl, pH 8. For agarose chromatography, running buffer: 0.05 M NaPO_4_, 0.15 M NaCl, pH 7.2.

### 4.2. Thioflavin T (ThT) Fluorescence Experiments

To evaluate the effect of quercetagitrin on tau aggregation in vitro, heparin sodium was used as the inducer, and ThT was used as the detection reagent. In brief, tau-K18 (50 μM), DTT (2 μM), heparin sodium (12.5 μM), ThT (60 μM), and different concentrations of quercetagitrin (0, 3.12, 6.25, 12.5, 25, 50, and 100 μM) were mixed in a 96-well plate and incubated at 37 °C in a microplate reader (Synergy H1, Biotek, Winooski, VT, USA), and the fluorescence value was monitored every half an hour for 48 h (λex: 440 nm, λem: 485 nm). Quercetagitrin was firstly dissolved in DMSO at 10 mM and then diluted to the working concentration with Tris-HCl buffer (50 mM Tris, 100 mM NaCl, pH 7.4). Other components were diluted with Tris-HCl buffer. DTT and protein were premixed in advance before use.

### 4.3. Transmission Electron Microscopy (TEM)

The effect of quercetagitrin on tau-K18 fibrilization morphologies was investigated by performing TEM. In brief, the protein samples from the end of the ThT fluorescence experiments were diluted to 5 μM with ddH_2_O and spotted onto a 230-mesh carbon-coated copper grid (BZ1102, EMCN, Beijing, China). After incubation for 30 min, the residual solution on the surface of the grid was removed by using filter paper. The grids were washed with ddH_2_O and mixed with 1% uranium acetate for 30 s. The morphology of tau-K18 fibrils was observed using TEM (JEM1230, Tokyo, Japan).

### 4.4. Animals and Drug Treatment

The P301S-tau transgenic mice expressing human P301S mutant 1N4R tau were purchased from the Jackson Laboratory. Adult C57BL/6 mice were purchased from the Guangdong medical laboratory animal center. In this study, P301S-tau transgenic mice were randomly assigned to drug or vehicle groups. For the drug groups, the drinking water contained 10 μg/mL quercetagitrin (HY-N4150, MedChemExpress LLC, Shanghai, China) and 0.05% DMSO (*v/v*). P301S-tau transgenic mice-vehicle group and age-matched C57 mice were given water containing 0.05% DMSO (*v/v*). Because of the photolysis of quercetagitrin, only 100 mL of the drug solution was given to the mice each time, and the container was wrapped with tinfoil to avoid light. All mice were housed in standard conditions with free access to food and water. All animal protocols were approved by the Ethics Committee of Shenzhen University in Animal Experimentation (permit number AEWC-20140615-002).

### 4.5. Behavioral Test

In this study, the mice’s movements were recorded using the Smart V3.0 computerized tracking system.

### 4.6. Y-Maze

The Y-maze apparatus used in this study consisted of three opaque arms spaced at angles of 120° from each other. The three arms were interconnected in the middle to form a Y shape. The experimental procedure was adapted from published protocols [31]. Briefly, each mouse was placed at the distal end of one of the arms and allowed to explore the maze freely for 5 min. The camera recorded the movement traces of each mouse. An alternation was defined as occurring when the mice entered three different arms in succession. The alternation rate (%) was calculated using the following formula: Alternation (%) = [Number of alternation/(Total number of arm entries-2)] × 100%.

### 4.7. Morris Water Maze (MWM)

In this study, the MWM test was conducted in a circular pool with a diameter of 160 cm and a depth of 50 cm. The pool was divided into four quadrants by two diameters perpendicular to each other. The pool was filled with opaque water up to 20 cm from the top of the wall. An escape platform was placed in one quadrant of the pool. The height of the escape platform was 1 cm below the surface of the water. The MWM test was divided into two stages: a training period and a testing period. In the training period, the mice were placed in the water at one quadrant without a platform, facing towards the wall of the maze. Then, they were allowed to explore the pool freely to find the platform. If the mice found the platform within 60 s, they could reside there for 15 s. The time taken to find the platform was recorded. If not, the mice were guided to the platform and resided there for 15 s. In this case, the time taken to find the platform was recorded as 60 s. The training process was repeated three times a day starting in different quadrants without the platform. The above training process was executed for four consecutive days, and the detection was performed 24 h after the last round of training. During the testing period, the hidden platform was removed, and the mice were placed into the pool at the quadrant opposite the platform. The mice were allowed to explore the pool freely, and their movements were recorded for one minute [32].

### 4.8. Cell Culture, Transfection, and Drug Treatment

Human embryonic kidney 293 (HEK293) cells were cultured in high-glucose DMEM supplemented with 10% fetal bovine serum (FBS) and 1% penicillin/streptomycin solution. Mouse neuroblastoma (Neuro2a, N2a) cells were cultured in the medium with 45% Opti-MEM, 50% DMEM, 5% FBS, and 1% penicillin/streptomycin solution. The cells were kept in a cell incubator containing 5% CO_2_ at 37 °C. Transfection of P301S-tau plasmids was performed when the cells reached 75% confluence using the LipoFectMax™ Transfection Reagent (FP310, ABP Biosciences, San Diego, CA, USA). At 24 h after transfection, the culture medium was changed to a fresh culture medium containing different concentrations of quercetagitrin and 0.01% DMSO. For HEK293 cells, 0, 10, 20, or 40 μM quercetagitrin was used, and for N2a cells, 0, 5, 10, or 15 μM quercetagitrin was used. The cells were collected using a cell scraper 24 h after the quercetagitrin treatment.

### 4.9. Cytotoxicity Testing

HEK293 cells or N2a cells were cultured in a 96-well plate. The culture medium was changed to a medium containing different concentrations of quercetagitrin (0, 2, 5, 10, 20, 30, 40, 60, 80, and 100 μM) with 0.1% DMSO when the cells reached 70–80% confluence. After 24 h, the culture medium was changed to a fresh medium containing 10% cck-8 reagent (MF128-01, Mei5bio, Beijing, China). After a 2-h incubation, the absorbance at 450 nm (Synergy H1, Biotek, Winooski, VT, USA) was measured, and the cell viability was calculated using the following formula: cell viability (%) = [(As − Ab)/(Ac − Ab)] × 100%. As refers to the absorbance of the sample containing cells, drug, and reagent; Ab is the absorbance of the blank control containing 10% cck-8 reagent in the absence of the cells and drug; and Ac is the absorbance of the negative control containing cells and 10% cck-8 reagent in the absence of the drug.

### 4.10. Protein Extraction

For tissue samples, mice hippocampus or cortex tissues were lysed in RIPA lysate (50 mM Tris, 150 mM NaCl, 1% Triton X-100, 1% sodium deoxycholate, and 0.1% SDS, pH 7.4) and homogenized by using a freeze homogenizer at 4 °C (65 Hz, 10 min). Protease phosphatase inhibitor (P002, NCM Biotech, Shanghai, China) was added into RIPA lysate at the ratio of 1:100 before use. The tissue homogenate was centrifuged at 12,000× *g* for 25 min at 4 °C. The supernatant was collected and sonicated for 1 min (15% ultrasound power, 2 s sonication, 2 s interval). For cell samples, the RIPA lysate was added to the cell culture wells after they were washed with PBS buffer once. The cells were scraped off using a cell scraper and collected in a sample tube. The samples were placed on ice for 30 min and then sonicated for 1 min (15% ultrasound power, 2 s sonication, 2 s interval). For both tissue samples and cell samples, protein concentrations were determined using a BCA assay kit (P0009, Beyotime, Shanghai, China) and homogenized with RIPA lysates.

### 4.11. Western Blotting

The protein samples in the loading buffer (50 mM Tris-HCL, 2% SDS, 10% glycerol, and 0.1% bromophenol blue) were boiled for 8 min at 95 °C. The proteins were dispersed by performing SDS-PAGE gel electrophoresis (PowerPac Basic, BioRad, Hercules, CA, USA). After electrophoresis, the proteins were transferred to nitrocellulose membrane from the SDSPAGE gels (DYCZ-40D, BioRad, USA). The membranes were blocked in blocking solution (Beyotime, P0252, Shanghai, China) for 20 min and then incubated with primary antibody at 4 °C overnight. The following primary antibodies were used in this study: AT8 (MN1020, Invitrogen, Carlsbad, CA, USA,1:1000), AT270 (MN1050, Invitrogen, Carlsbad, CA, USA, 1:1000), AT180 (MN1040, Invitrogen, Carlsbad, CA, USA, 1:1000), AT100 (MN1060, Invitrogen, Carlsbad, CA, USA, 1:1000), Tau13 (835201, Biolegend, San Diego, CA, USA, 1:1000), Actin (3700S, CST, Danvers, MA, USA, 1:1000), GAPDH (ab8245, abcam, Cambridge, UK, 1:1000), Ace-tau (K174) (HW181, SAB, Los Angeles, CA, USA, 1:1000), T22 (ABN45S, EMD Millipore, Darmstadt, Germany), PP2A (2259T, CST, Danvers, MA, USA), GSK3β (93926, abcam, Cambridge, UK, 1:1000), p-GSK3β (75745, abcam, Cambridge, UK, 1:1000), NF-κB (8242S, CST, Danvers, MA, USA, 1:1000), p-NF-κB (3033S, CST, Danvers, MA, USA, 1:1000), IL1α (50794S, CST, Danvers, MA, USA, 1:1000), and IL6 (12912S, CST, Danvers, MA, USA, 1:1000). The membrane was washed two times with TBST buffer and incubated with horseradish peroxidase (HRP)-conjugated secondary antibody for 1 h at room temperature. The secondary antibodies were diluted in TBST buffer at a ratio of 1:1000. At the end, the membrane was washed three times with TBST buffer at room temperature. Immunoreactive bands were visualized using an enhanced chemiluminescence kit (P0018FM, Beyotime, Shanghai, China). The image was then imported into ImageJ software to detect the grayscale values of the bands. To compare the relative expression levels between different samples, the grayscale values of the target protein were divided by the grayscale values of the loading control. Then, the values were imported into GraphPad Prism 8.0 for the analysis of significant differences.

### 4.12. Immunohistochemistry Staining

For immunohistochemical staining, a polymer-based detection kit (PV6000, ZSGB-BIO, Beijing, China) was used. In brief, mouse brain slices were immersed in endogenous peroxidase blocker for 10 min after being washed three times in PBS. Then, the slices were mounted onto slides and incubated with primary antibody (AT8, MN1020, Invitrogen, Carlsbad, CA, USA, 1:200) overnight. The slices were washed three times in PBS and incubated with HRP-conjugated secondary antibody for 20 min at 37 °C. A DAB Concentrate Bulk Kit (ZLI9017, ZSGB-BIO, Beijing, China) was used for chromogenic reaction. The slices were immersed in DAB chromogenic agent for 8 min followed by being washed three times in ddH2O. The slides were dehydrated in 50%, 75%, and 95% ethanol, successively, for 8 min each time. The slides were transferred into xylene and sealed with PerMount mounting medium. Histochemical staining images were visualized using a pathological slice scanner (Aperio CS2, LEICA, Wetzlar, Germany). The images were processed using ImageJ to quantify the staining intensity and area. The quantitative analysis method can be summarized as follows: the area and grayscale value of the hippocampus and cortical regions were detected from each brain slice, and the proportion of positively stained areas was calculated. Then, the values were imported into Graph Prism 8.0 for *t*-test analysis of significant differences.

### 4.13. Immunofluorescence Staining

After being washed three times with PBS, the brain slices were immersed in Triton-X100 for 15 min and then washed twice with PBS. After that, the slices were blocked in blocking solution for 20 min at room temperature and incubated with primary antibody overnight at 4 °C. The slices were washed three times in PBS, then incubated with Alexa Fluor488/555-conjugated isotype-specific secondary antibody (A0453/A0423, Beyotime, Shanghai, China) for 1 h at 37 °C followed by DAPI staining for 8 min at room temperature. The slices were mounted onto slides after being washed three times in PBS. Labeled sections were visualized using a confocal microscope (LSM880, Zeiss, Oberkochen, Germany). The statistical analysis procedure for this section was similar to that used for the immunohistochemistry staining. The following primary antibodies were used for immunofluorescence in this study: GFAP (ab5541, abcam, Cambridge, UK, 1:500), Iba1(019-19741, Wako, Osaka, Japan, 1:200), NeuN (ab104224, abcam, Cambridge, UK,1:500), and synaptophysin (ab32127, abcam, Cambridge, UK, 1:100).

### 4.14. Thio-S Staining

Thio-S was used to fluorescently label tau aggregates with a β-sheet structure. In brief, after being washed three times in PBS, the brain slices were incubated in 0.3% Thio-S (Sigma, Darmstadt, Germany, dilution in 50% ethyl alcohol) followed by being washed three times in 50% ethyl alcohol and PBS, respectively. Then, the slices were mounted onto slides and visualized using a confocal microscope (LSM880, Zeiss, Oberkochen, Germany). The statistical analysis procedure for this part was similar to that used for the immunohistochemistry staining.

### 4.15. Statistical Analysis

Results were shown as mean ± SEM. Statistical analysis was performed using GraphPad Prism 8.0.2 statistical software. Statistical significance was assessed using Student’s *t* test, with one-way or two-way ANOVA. *p* values of 0.05 or less were considered to denote significance. All the statistical analysis details were summarized in Table S1.

## Data Availability

The data that support the findings of this study are available from the corresponding author upon reasonable request.

## Supplementary Materials

The following supporting information can be downloaded at: https://www.mdpi.com/article/10.3390/molecules28093964/s1.

## Abbreviations

AD: Alzheimer’s disease; DTT: dithiothreitol; GFAP: glial fibrillary acidic protein; GSK3β: glycogen synthase kinase 3β; HEK293: human embryonic kidney 293; Iba1: Ionized calcium binding adapter molecule 1; IL1α: interleukin1α; IL6: interleukin6; IFN-γ: interferon-γ; MWM: Morris water maze; NFTs: neurofibrillary tangles; N2a: Neuro2a; NF-κB: nuclear factor kappa-B; PHFs: paired helical filaments; PP2A: phosphatase 2A; ThT: thioflavin T; TNF-α: tumor necrosis factor; TEM: transmission electron microscopy.
