# Supplementary material for: Quercetagitrin Inhibits Tau Accumulation and Reverses Neuroinflammation and Cognitive Deficits in P301S-Tau Transgenic Mice
Source: Molecules. 2023 May 8;28(9):3964. doi: 10.3390/molecules28093964 (PMC10180163; doi:10.3390/molecules28093964)
Supplement: Supplementary file 1 [file molecules-28-03964-s001.zip › Figure S1.pdf]

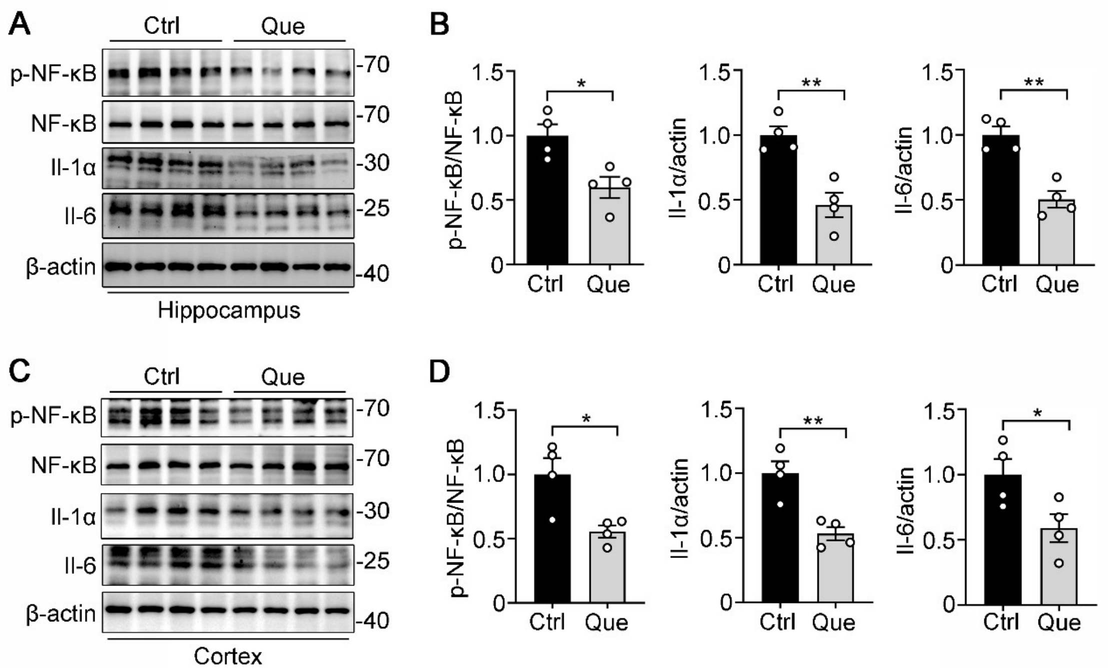

Supplementary Figure S1 Quercetagitrin blocks NF-κB activation and inflammatory responses in P301S-tau transgenic mice

(A) Western blot analysis of p-NF-κB (S468), total NF-κB, Il-1*α*, Il-6 and β-actin in the hippocampus of control and quercetagitrin-treated P301S-tau transgenic mice. (B) Quantification of p-NF-κB normalized to total NF-κB, Il-1*α* and Il-6 normalized to β-actin. (C) Western blot analysis of p-NF-κB (S468), total NF-κB, Il-1*α*, Il-6 and β-actin in the cortex of the mice in three groups. (D) Quantification of p-NF-κB normalized to total NF-κB, Il-1*α* and Il-6 normalized to β-actin. Data were expressed as mean ± SEM, $^{*}p < 0.05$, $^{**}p < 0.01$.
